# The Determination of a Consensus Nutritional Approach for Cancer Patients in Spain Using the Delphi Methodology

**DOI:** 10.3390/nu14071404

**Published:** 2022-03-28

**Authors:** José Pablo Suárez-Llanos, Ruth Vera-García, Jorge Contreras-Martinez

**Affiliations:** 1 Clinical Nutrition and Dietetics Unit, Hospital Universitario Nuestra Señora de Candelaria, 38010 Santa Cruz de Tenerife, Spain; 2 Medical Oncology Department, Hospital Universitario de Navarra, Irunlarrea 3, 31008 Pamplona, Spain; rveragar@navarra.es; 3 Radiation Oncology Department, Hospital Regional de Málaga, 29009 Málaga, Spain; jorgecontrerasmartinez@gmail.com

**Keywords:** Delphi methodology, nutritional condition, nutritional therapy, oncology, sarcopenia, screening

## Abstract

Malnutrition has a multifactorial origin and can be caused by cancer. This study determined the consensus of a panel of experts on the nutritional approach for cancer patients in Spain using a multidisciplinary approach. Using the Delphi methodology, a 74-question questionnaire was prepared and sent to 46 experts. The areas of knowledge addressed were the nutritional status of the cancer patient, nutritional screening, nutritional therapy, patient referral, and multidisciplinary care. A total of 91.7% of the experts agreed with the questions posed on nutritional status, 60.0% with those on nutritional screening, 76.7% with those on nutritional therapy, and the entire panel of experts agreed with the questions posed on patient referral and multidisciplinary care. The experts agreed upon a high prevalence of malnutrition among cancer patients in Spain. Unlike medical and radiation oncologists, medical nutrition specialists believe that body composition assessment should not be carried out in all types of cancer patients during nutritional screening and that interventions can be conducted outside the oncology clinic. In general, it is recommended that nursing staff routinely perform nutritional screening before starting cancer treatment. It is necessary to develop a multidisciplinary action protocol that includes nutritional and/or sarcopenia screening.

## 1. Introduction

Malnutrition has a multifactorial origin and can be caused by inadequate dietary intake (e.g., due to decreased appetite sensation as a result of changes in cytokines, glucocorticoids, insulin, or growth factors), reduced absorption of nutrients (e.g., in patients with intestinal or pancreatic insufficiency), or an increase in nutrient requirements (e.g., enterocutaneous burns or fistulas) [1,2]. Malnutrition associated with cancer and the side effects of therapies [3] becomes a common complication that can negatively affect the outcome of treatment and the patient’s quality of life and result in unscheduled hospitalizations and decreased survival [4,5,6,7].

Approximately 72.5% of cancer patients experience eating problems during the course of their disease, and overall, 69.6% experience weight loss after diagnosis [3]. In the PREDyCES [8] and SeDREno [9] studies, malnutrition prevalences of 33% and 39%, respectively, were observed in cancer patients at hospital admission. This malnutrition is related to an increase in morbidity, a loss of muscle mass [10], more extended hospital stay, poorer quality of life, higher economic expenditure [11], and an increase in mortality in up to 20% of patients [12,13].

On the other hand, sarcopenia consists of a decrease in muscle mass associated with a reduction in the functional capacity of the patient, which in the case of cancer can cause a significant dependence to carry out activities of daily living and can become a hardly reversible situation. This is due to the secretion of proinflammatory cytokines and tumor factors that cause metabolic alterations, systemic inflammation, and decreased appetite [13]. In the absence of specific pharmacological treatment, the multifocal approach to cancerous sarcopenia with dietary-nutritional management constitutes the central axis of therapy and can improve clinical outcomes [14].

Personalized nutritional interventions can be beneficial by offering cancer patients advice on oral intake accompanied and, if necessary, by oral nutritional supplements (ONSs). If oral intake is insufficient or unfeasible, enteral tube nutrition should be considered, or parenteral nutrition should ultimately be considered in patients with contraindications for the former [15,16]. There is significant evidence demonstrating the importance of nutritional support as a valuable measure in the general oncology strategy [17,18].

However, even knowing the importance of nutritional status in the prognosis and quality of life of cancer patients and the benefits of nutritional treatment, screening for malnutrition and/or sarcopenia is not widespread in oncology units. This should be carried out from the moment of diagnosis and periodically during the clinical evolution of the oncological process [2].

This study aimed to reach a consensus on the nutritional approach for cancer patients in Spain from a multidisciplinary approach.

## 2. Materials and Methods

The Delphi methodology was used based on a group facilitation technique that allows the opinions to be transformed into a group consensus [19].

The panel of experts was chosen based on the following criteria: five or more years of experience in their specialty, interest in nutrition, and at least one scientific publication in the area of nutrition. These experts were invited to participate in the consensus through questionnaires by email. The steering committee and the members of the panel did not know the identity of the respondents. The panel initially consisted of 46 experts: 15 medical nutrition specialists, 15 medical oncologists, and 16 radiation oncologists.

A literature review was conducted to identify gaps in evidence supporting the content of the survey. The steering committee led the development of the surveys for each round of voting, reviewed the responses and created response summaries, validated the systematic literature search, and critically evaluated the evidence. A total of 74 initial questions were asked that were distributed among the following blocks of knowledge: (1) the nutritional status of the cancer patient, (2) nutritional screening, (3) nutritional therapy, and (4) patient referral and multidisciplinary care.

Two rounds were conducted with the experts: the first between 18 May and 22 June, 2021, and the second between 14 July and 27 August, 2021. The questions were anonymous and were answered through online questionnaires.

The study was based on a survey and did not involve the participation of human subjects or the management of patient data, nor was it intended to modify the current clinical practice of the participants. Consequently, this study did not require ethical approval.

### Statistical Analysis

The questions with discrete quantitative answers for each item were evaluated using a Likert scale from 0 to 11 points (0 = completely disagree; 10 = completely agree). The consensus criterion used was as follows: for agreement, a median ≥ eight and an interquartile range (IQR) <0.4; for disagreement, a median ≤ two and an IQR <0.4. Consensus was obtained for the questions with nominal categorical answers when one of the answers reached at least 50% of the total mentions.

The items were evaluated as a whole by medical specialists (i.e., medical nutrition specialists, medical oncologists, and radiation oncologists). Nonparametric tests were used to determine whether there were significant differences among the specialist groups. For the questions with a nominal categorical response, the chi-square test was performed. In the questions with a discrete quantitative response, the Levene test was performed to ensure the assumptions of the application of the Kruskal–Wallis test. If significant differences were found, the Mann–Whitney test with the Bonferroni correction was applied.

For all tests, the level of significance was *p* ≤ 0.05. Data were analyzed using Gandia Barbwin version 7.0.2110.5(Tesi S.L., Gandia, Valencia, Spain) and XLSTAT^®^ version 21.04 (Addinsoft SARL, Paris, France) of Microsoft Excel^®^.

## 3. Results

### 3.1. Nutritional Condition

The experts agreed that there is a high prevalence of malnutrition among cancer patients in Spain and that the assessment of their nutritional status should be a priority. This information should be included as part of the clinical data before starting any treatment. Experts agree that it must be taken into account that physical exercise and the patient’s functional capacity are related to nutritional status must be taken into account. Furthermore, there is a direct relationship between weight maintenance and the patient’s muscle mass.

**Figure 1:**
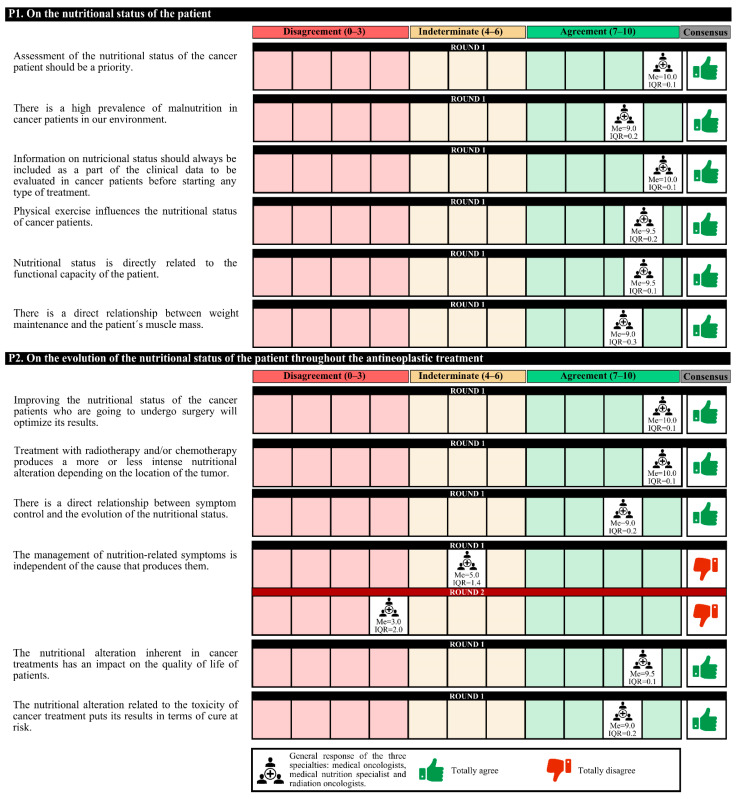
Answers about nutritional status. IQR, interquartile range; Me, median.

On the other hand, experts agree that improving the nutritional status of cancer patients who undergo surgery could optimize the results. The following should be taken into account: treatment with radium and/or chemotherapy produces nutritional alterations depending on the location of the tumor; there is a direct relationship between symptom control and the evolution of nutritional status; and nutritional alterations have an impact on the quality of life of patients that jeopardizes the results of the treatment in terms of cure. In the first round, the degree of agreement was 91.7%. In the second round, no final consensus was obtained (Figure 1).

### 3.2. Nutritional Screening

The experts agreed that nursing staff should routinely perform nutritional screening before initiating cancer treatment, as it enables more effective nutritional therapy. The screening tool must be simple, fast, and specific, and the Malnutrition Universal Screening Tool (MUST) may be a suitable option. It is also necessary to perform simple sarcopenia screening and functionality tests associated with nutrition.

In case of a lack of time to carry out the screening, experts agree that the screening could be carried outu remotely by a health care professional. A total of 58.7% of the experts considered that the most appropriate outpatient nutritional screening characteristic to implement in the oncology unit is feasible, with simple variables and short duration, although it is less sensitive and specific.

**Figure 2:**
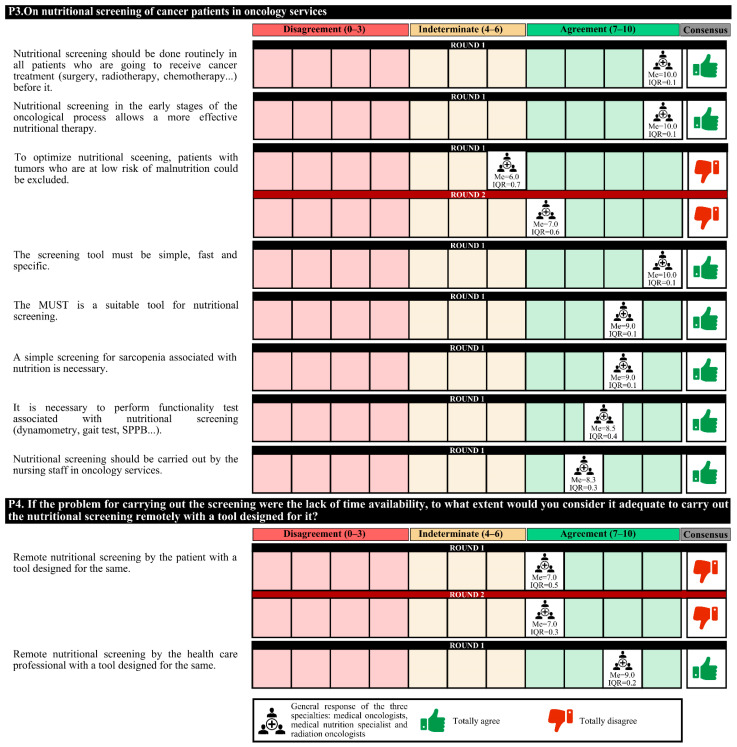
Answers about nutritional screening (I). IQR, interquartile range; MUST, Malnutrition Universal Screening Tool; Me, median; SPPB, short physical performance battery.

**Figure 3:**
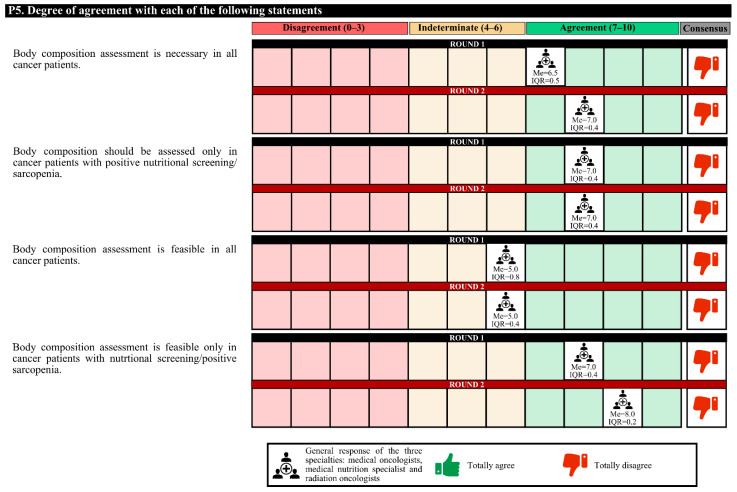
Answers about nutritional screening (II). IQR, interquartile range; Me, median.

**Figure 4:**
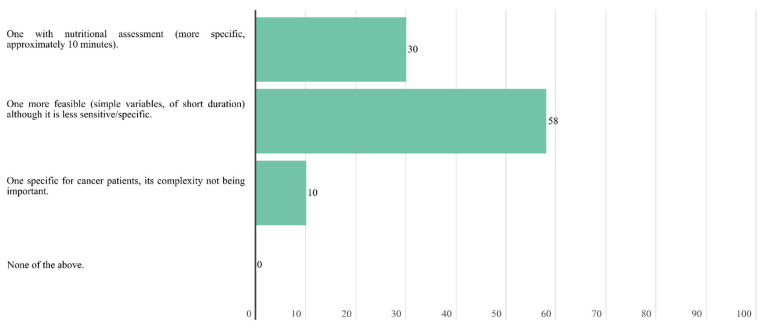
Answers to the question “What type of outpatient nutritional screening do you see as the most appropriate to implement in the medical/radiation oncology unit of your hospital?”.

In the first round, the degree of agreement was 60.0%, and in the second round, the experts agreed only that body composition assessment is required only in cancer patients with positive nutritional or sarcopenia screening (Figure 2, Figure 3 and Figure 4).

### 3.3. Nutritional Therapy

The experts agreed that nutritional therapy and cancer treatment should be simultaneous, taking into account certain times to scale the nutritional intervention according to the disease evolution of the patient. Patients with positive nutritional screening should be given dietary recommendations and ONSs, with recommendations to perform physical exercise (if appropriate). Agreement among medical nutrition specialists was less than that among medical and radiotherapy oncologists that these interventions should be carried out in the oncology consultation.

Prophylactic gastrostomy placement is recommended in patients with head and neck cancer with impaired swallowing, regardless of their nutritional status. In these patients and those treated for digestive tumors, experts recommended the perisurgical use of immunomodulatory formulas.

The initiation of empiric use of pancreatic enzymes after partial or total pancreatectomy is recommended. However, 63.0% of experts considered that in patients with grade IV oral and/or esophageal mucositis that prevents proper swallowing, the most appropriate therapeutic option is transitory parenteral nutrition.

Regarding the characteristics of the nutritional formulas, for patients with postsurgical feeding jejunostomies due to digestive tumors, these formulas must be of low viscosity and osmolarity, have high energy density and protein content and be in 1000 cc containers to avoid night replacement. For patients discharged with ileostomy, the nutritional formula characteristics that should be met are low osmolarity, easily digestible, and high energy density and protein content.

Experts agree that in cancer patients, it is essential to assess whether the nutritional formula has a low glycemic index, a high energy density and protein content, and fiber, as well as the omega-3 fatty acid (in patients with cachexia) and slow-release carbohydrate content. For cancer patients with a loss of muscle mass, it is important to assess the high energy density and protein content and whether hydroxymethyl butyrate (HMB) and vitamin D are present. For cancer patients with digestive disorders, it is important to assess the high energy density and hydrolyzed protein content and whether medium-chain triglycerides (MCTs) and various flavors are present.

In the first round, the degree of agreement was 76.7%. In the second round, the experts finally agreed on the placement of a prophylactic gastrostomy in patients with head and neck cancer with obvious malnutrition who are to undergo surgery and/or chemotherapy; the empiric use of pancreatic enzymes in all patients with advanced pancreatic cancer; and, for patients with postsurgical feeding jejunostomies due to digestive tumors, the importance of the use of nutritional formulas that do not contain fiber.

**Figure 5:**
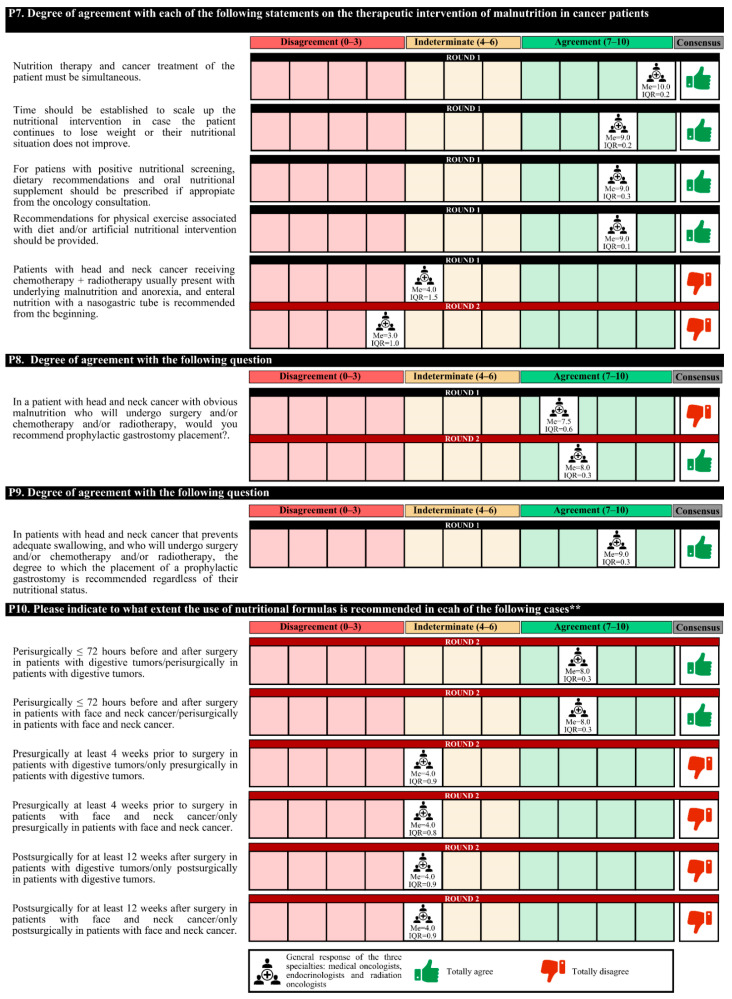
Answers about nutritional therapy (I). ** The rephrased questions for the second round are shown due to a lack of understanding in the first round. IQR, interquartile range; Me, median.

**Figure 6:**
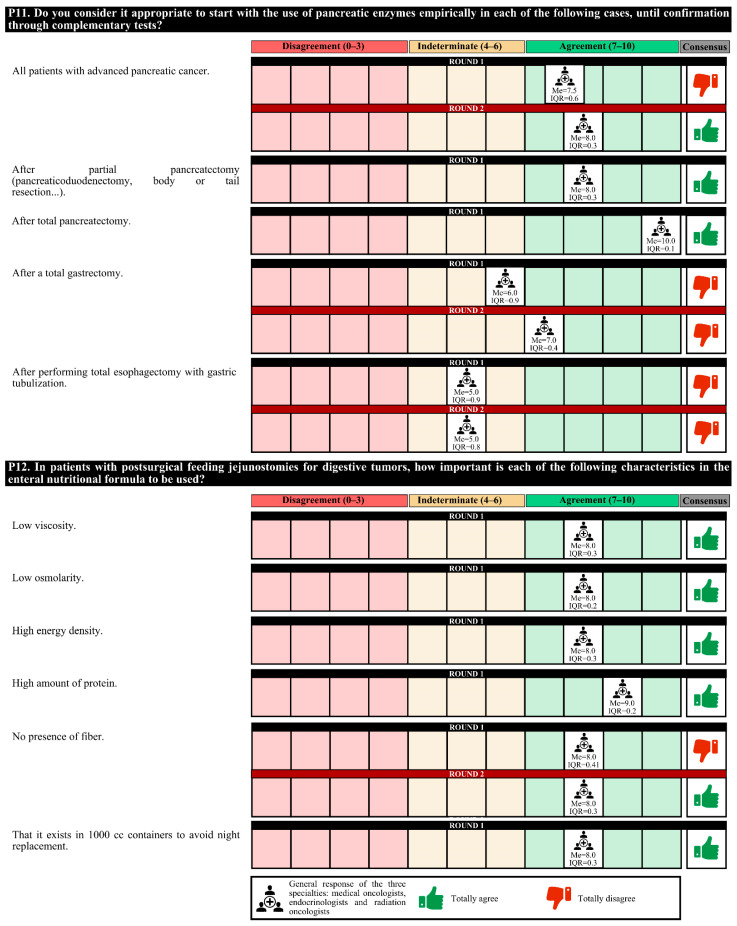
Answers about nutritional therapy (II). IQR, interquartile range; Me, median.

**Figure 7:**
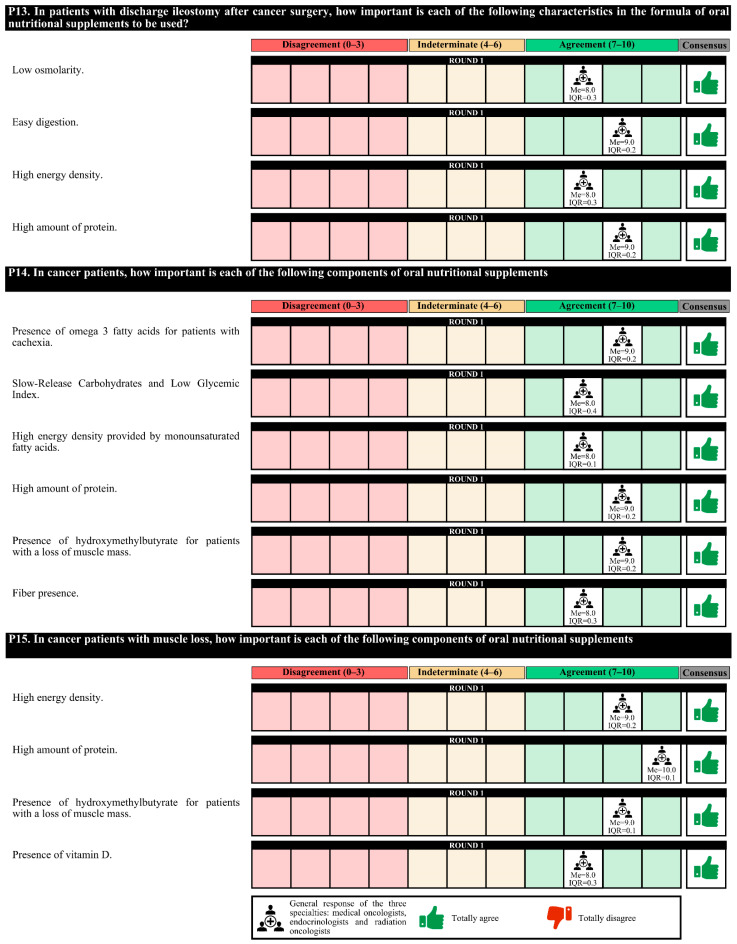
Answers about nutritional therapy (III). IQR, interquartile range; Me, median.

**Figure 8:**
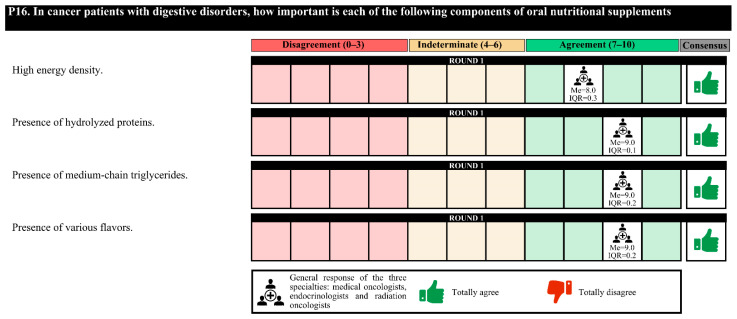
Answers about nutritional therapy (IV). IQR, interquartile range; Me, median.

**Figure 9:**
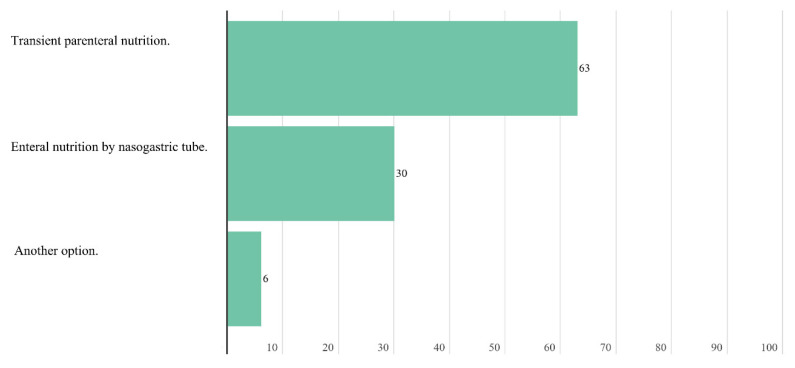
Answers to the question “In patients with already established grade IV oral and/or esophageal mucositis that prevents adequate swallowing, what nutritional therapeutic option would you choose?”.

In the first round, confusion was detected in the questions regarding the nutritional formulas, and these questions were redrafted for the second round. The experts agreed only on the perisurgical use of immunomodulatory formulas in patients with digestive or head and neck tumors (Figure 5, Figure 6, Figure 7, Figure 8 and Figure 9).

### 3.4. Patient Referral and Multidisciplinary Care

**Figure 10:**
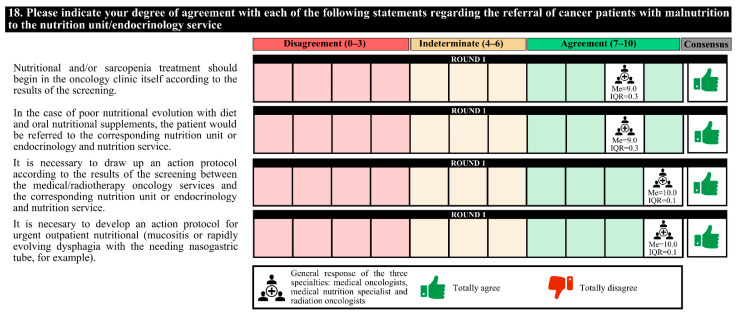
Answers on patient referral and multidisciplinary care. IQR, interquartile range; Me, median.

All experts agreed that nutritional and/or sarcopenia treatment should be started in the oncology clinic itself based on the screening results. In the case of poor nutritional evolution, the patient would be referred to the corresponding nutrition unit. It is necessary to develop an action protocol according to the screening results and to urgently initiate outpatient nutritional treatments. The degree of agreement was 100% (Figure 10).

### 3.5. Valuation between Specialties

In the first round, medical nutrition specialists disagreed with radiation oncologists (neutral opinion) that the assessment of the body composition of all cancer patients is feasible. In the second round, both specialties had a neutral opinion. The same happened in the aspect that assessing the body composition only of cancer patients with positive nutritional screening/sarcopenia is feasible. In the second round, both specialties agreed.

In the second round, the opinions of the specialists differed for only one approach. Medical nutrition specialists agreed that screening patients with a remote tool was appropriate, whereas medical oncologists had a neutral opinion.

**Figure 11:**
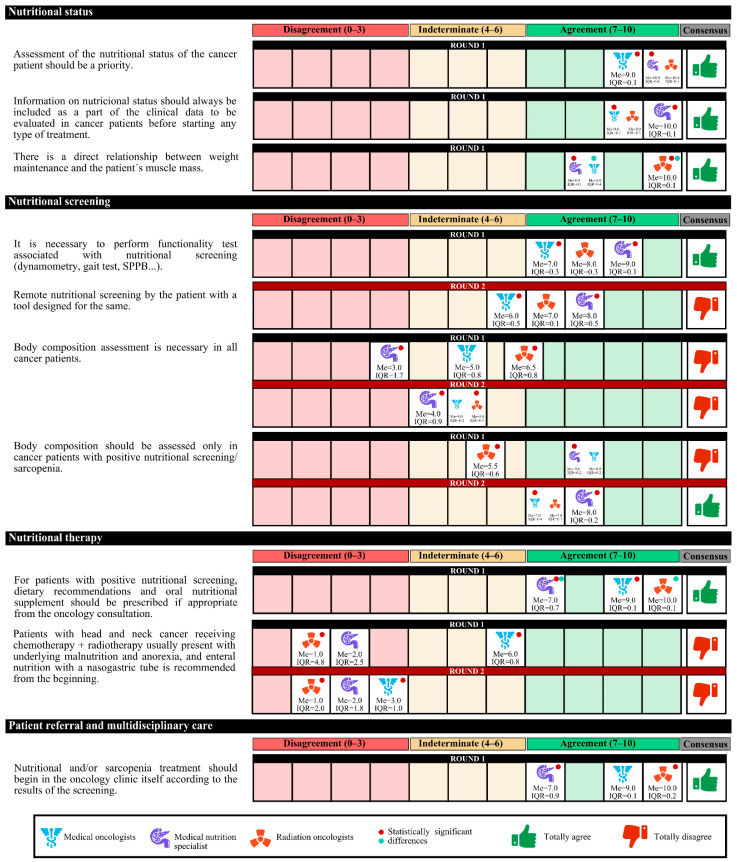
Responses with significant differences among the different medical specialties. All tests had a *p* value < 0.05. IQR, interquartile range; Me, median. SPPB, short physical performance battery.

Figure 11 shows the statistically significant differences in the responses among the different specialists.

## 4. Discussion

This survey is one of the first carried out in Spain in which the nutritional management of cancer patients is addressed. The general results showed a high degree of agreement among the experts.

In general, medical oncologists, radiation oncologists, and medical nutrition specialists specialized in nutrition agreed that there is a high prevalence of malnourished cancer patients, and although this incidence varies greatly depending on the type and location of tumor and cancer stage, this condition affects the results of treatments and negatively impacts patients’ clinical course, as well as their quality of life. Several studies link malnutrition with an increase in the morbidity and mortality of cancer patients, among them the recently published SeDREno study in Spain [9], where almost 40% of cancer patients were malnourished, and cancer patients had a more than 50% risk of developing malnutrition than noncancer patients [20,21,22,23]. The nutritional management of these patients involves different medical specialists (mainly medical and radiotherapy oncologists and medical nutrition specialists). Therefore, nutritional management should be coordinated from a multidisciplinary point of view.

Although detection systems based on artificial intelligence have been developed in recent years [24], experts continue to recommend as a goal of personalized medicine that screening be performed by nursing staff or remotely by a health professional. It should be noted that the dynamics of including screening by default in patient care should not be exclusive to the nursing staff and can be extended to any other health care professional. Therefore, it is important to highlight the need for individual training and make it known among each health care professionals.

Concerning nutritional assessment tools, there are many screenings with different characteristics (duration, prognostic capacity, etc.), but none is considered a reference or gold standard [25]. The panel expert recommends the MUST as a feasible and appropriate option. This screening shows the best correlation in general with the recent GLIM malnutrition criteria [26], including the cancer patient [27]. Therefore, whether or not this is implemented (which initially includes nutritional screening), the MUST seems to detect cancer patients at risk of malnutrition adequately. However, the lack of time and the care of many patients have not favored the general implementation of these screening tools.

Parallel to the global nutritional status of cancer patients, body composition and the importance of muscle mass and its relationship with the functional capacity of these patients are becoming increasingly important, as they are key aspects both in the clinical evolution of a patient’s disease and in a patient’s physical and mental health [28]. In recent years, the relationship between malnutrition and sarcopenia has become more relevant (in fact, the GLIM criteria themselves introduce a decrease in muscle mass as a criterion for malnutrition). Therefore, simple sarcopenia screening, such as the SARC-F (recommended by the European Working Group on Sarcopenia in Older People (EWGSOP) [29]), coupled with malnutrition screening, could be a handy tool in the global assessment of patients, as seen in the responses of experts.

In this consensus, medical nutrition specialists, medical oncology, and radiation oncology were selected. The specialty that showed the most differences in agreement from other specialties was medical nutrition specialists. The questions with less consensus were very oriented toward a particular nutritional therapy, where medical nutrition specialists have more knowledge and experience, while the questions with more consensus were more general. These findings influence the need to develop and implement training plans for specialists in cancer patients’ nutritional management.

To date, health professionals have been made aware of the impact that nutritional status has on cancer patients, but is necessary to implement tools for simple and feasible malnutrition and/or sarcopenia screenings, protocols for action based on screening results and for specific training for healthcare personnel, and to cooperate through multidisciplinary work, referring the patient to specialized units in the case of poor nutritional evolution.

## 5. Conclusions

The experts agreed upon a high prevalence of malnutrition among cancer patients in Spain. Unlike oncologist and radiation oncologists, medical nutrition specialists believe that body composition assessment should not be carried out in all types of cancer patients during nutritional screening and that interventions can be conducted outside the oncology clinic. In general, it is recommended that nursing staff routinely perform nutritional screening before starting cancer treatment. It is necessary to develop a multidisciplinary action protocol that includes nutritional and/or sarcopenia screening. 

## Data Availability

The data presented in this study are available on request from the corresponding author.
